# Hospital Meetings

**Published:** 1904-07-02

**Authors:** 


### HOSPITAL MEETINGS.

### FRIEDENHEIM HOSPITAL (HOME OF PEACE FOR THE DYING), SWISS COTTAGE, N.W.

The annual meeting of Friedenheim Hospital, Swiss Cottage, N.W., took place last Saturday in the adjoining lecture hall of the School for the Blind, and was well attended. The chair was taken by the Mayor of Hampstead (Dr. Collingwood Andrews), and the reports showed that during the last year 150 patients had been admitted, and that the number of beds and cots in the hospital was now increased to 48. Owing chiefly to an erroneous report having been circulated that Friedenheim had received a large legacy, the financial condition of the hospital left much to be desired. There was a debt of £1,100 on the building fund, and a deficit of £55 15s. 11d. on the general fund.

The Mayor, in proposing the adoption of the reports, expressed his profound conviction of the need of such a home, where the dying received spiritual as well as material aid. He spoke highly of the admirable arrangements of the institution, and the spirit in which it was managed, drawing particular attention to the devotion of Miss Davidson, the foundress and hon. superintendent.

Mr. J. E. Mathieson seconded the resolution, which was then carried.

Mr. John Langton, in proposing the election of two new members of the Council, Mr. C. Earl and Dr. Clark Wilson, remarked that Friedenheim does not enter into competition with poor-law infirmaries, but is intended for a different class of persons. The cases received were chiefly cancer and consumption.

This resolution having been adopted, and a vote of thanks accorded to the medical staff, Dr. Lush, in moving a vote of thanks to the chartered accountants, spoke of some of the cases in the hospital, and said that there was not always sadness at Friedenheim, and that very few children die there.

Dr. Schofield, in seconding the last resolution, which was carried unanimously, appealed for drawing-room meetings as a means of helping the institution.

A hearty vote of thanks to the chairman then terminated the proceedings.

---

We have been requested by the council of the Friedenheim Hospital to correct an erroneous report to which considerable publicity has apparently been given during the past months. The hospital, the council state, unfortunately has not recently received a large legacy, as has been stated, and its many friends who have written congratulations are assured that their support is still as greatly needed as ever.

